# Global change, parasite transmission and disease control: lessons from ecology

**DOI:** 10.1098/rstb.2016.0088

**Published:** 2017-03-13

**Authors:** Joanne Cable, Iain Barber, Brian Boag, Amy R. Ellison, Eric R. Morgan, Kris Murray, Emily L. Pascoe, Steven M. Sait, Anthony J. Wilson, Mark Booth

**Affiliations:** 1 School of Biosciences, Cardiff University, Cardiff CF10 3AX, UK; 2 Department of Neuroscience, Psychology and Behaviour, University of Leicester, Leicester LE1 7RH, UK; 3 The James Hutton Institute, Invergowrie, Dundee DD2 5DA, UK; 4 School of Veterinary Sciences, University of Bristol, Bristol BS40 5DU, UK; 5 Grantham Institute – Climate Change and the Environment, Faculty of Natural Sciences, Imperial College London, Exhibition Road, London SW7 2AZ, UK; 6 Department of Biodiversity and Molecular Ecology, Centre for Research and Innovation, Fondazione Edmund Mach, Via E. Mach 1, 38010 S. Michele all'Adige, Trentino, Italy; 7 School of Biology, University of Leeds, Leeds LS2 9JT, UK; 8 Vector-borne Viral Diseases Programme, The Pirbright Institute, Ash Road, Pirbright, Woking GU24 0NF, UK; 9 School of Medicine, Pharmacy and Health, Durham University, Durham TS17 6BH, UK

**Keywords:** infectious disease, climate change, sustainable control, stressors

## Abstract

Parasitic infections are ubiquitous in wildlife, livestock and human populations, and healthy ecosystems are often parasite rich. Yet, their negative impacts can be extreme. Understanding how both anticipated and cryptic changes in a system might affect parasite transmission at an individual, local and global level is critical for sustainable control in humans and livestock. Here we highlight and synthesize evidence regarding potential effects of ‘system changes’ (both climatic and anthropogenic) on parasite transmission from wild host–parasite systems. Such information could inform more efficient and sustainable parasite control programmes in domestic animals or humans. Many examples from diverse terrestrial and aquatic natural systems show how abiotic and biotic factors affected by system changes can interact additively, multiplicatively or antagonistically to influence parasite transmission, including through altered habitat structure, biodiversity, host demographics and evolution. Despite this, few studies of managed systems explicitly consider these higher-order interactions, or the subsequent effects of parasite evolution, which can conceal or exaggerate measured impacts of control actions. We call for a more integrated approach to investigating transmission dynamics, which recognizes these complexities and makes use of new technologies for data capture and monitoring, and to support robust predictions of altered parasite dynamics in a rapidly changing world.

This article is part of the themed issue ‘Opening the black box: re-examining the ecology and evolution of parasite transmission’.

## 1. Introduction

The current epoch of ecological time is driven by human interference [1]. Multiple anthropogenic stressors—including climate change, pollution, ocean acidification, habitat loss and fragmentation, urbanization, agricultural expansion and intensification, together with other changes in the use of water and land resources—are directly or indirectly impacting all species on earth (e.g. [2–5]). These changes may lead to the crossing or corrosion of critical thresholds, or ‘planetary boundaries’ ([6,7], see glossary), that induce physiological stress or complete system dysfunction, with negative consequences for individuals, populations and species. Such processes will have significant impacts on parasite natural history and infectious disease risks.

The anticipation of global change is not currently reflected in programmes of intervention against parasites of humans—instead the emphasis is on identifying vulnerable communities from retrospective data, and targeting those communities for intervention. In an attempt to synthesize and implement cost-effective interventions against the neglected tropical diseases (NTDs), there has been a concerted effort to distribute human medicines through mass drug administration (MDA) programmes in areas of high transmission [8], aided by donations from large pharmaceutical companies. These MDA campaigns rely largely on the presumptive treatment of putatively exposed individuals in ‘at risk’ populations [9]. The expectation, translated from the outputs of mathematical models, is that repeated MDA will reduce the size of the parasite population and simultaneously reduce levels of morbidity attributable to infection [9].

Such intervention programmes are possible because of developments in our understanding of the life cycles and ecology of parasites affecting humans and livestock, primarily gathered in the Victorian era [10]. Early optimism among health practitioners in wealthy countries that control and intervention strategies would eradicate infectious diseases continued up until the middle of the twentieth century (reviewed by [11]); yet despite early (and enduring) optimism, relatively little success, at least in terms of eradication, has been achieved. Among the NTDs, only Guinea worm is scheduled for eradication (most probably because only low-tech solutions are necessary to interrupt the transmission cycle; see §3).

Recent analysis of NTDs in Africa suggests, at first glance, that the MDA strategies have succeeded in reducing the number of infections. Where pharmaceutical interventions have had a clear effect in reducing infectious disease prevalence, the challenge now is to ensure that such success is sustainable in the context of environmental change. River blindness, caused by the nematode *Onchocerca volvulus* carrying the *Wolbachia* bacterium [12], was introduced into South America by the *Simulium* black fly, which has been treated with periodic ivermectin administration since 1991. Although it is unlikely that river blindness will ever be eradicated globally, prevalence has fallen from 50% to 4% of those at risk in the endemic population [13]; however, the implications of environmental changes for the long-term efficacy of this and other treatment programmes are not well understood.

Elsewhere, problems remain in terms of attributing causality to, or quantifying the success of, MDA programmes [8]. First, the historical data are imprecise and patchy; diagnosis of some infections has been characterized for decades by a lack of sensitive and/or specific tools [14]. Second, global climate models reveal an ever-changing pattern of land surface temperature, rainfall and vegetation cover across the surface of the planet [15]. Thus, contemporaneous environmental changes could potentially confound the effects of MDAs. Third, host range shifts may spread parasites into areas where monitoring and MDA are not being applied. Finally, the programmes themselves may have generated selection pressures, as has been observed in other systems such as malaria [16], leading potentially to resistance, adaptation and other evolutionary consequences.

**Figure 1.**
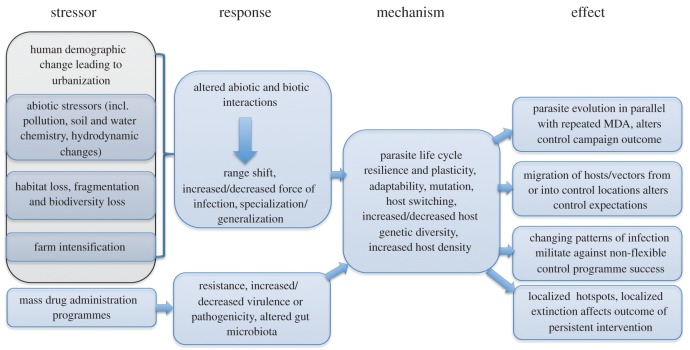
Stress–response impacts on parasite control programmes. (Online version in colour.)

In looking to the future sustainability and success of MDA and other interventions, we posit that it is imperative to consider what factors related to global change not only impinge on current efforts, but how global changes, including those brought about by control efforts themselves, might influence the outcome of attempts at control, elimination or eradication of specific infections. Figure 1 shows the hypothesis that a combination of stressors, brought about by global anthropogenic change, will induce a set of responses that have a significant impact on control prospects of NTDs in particular and parasites of economic importance in general.

Given the paucity of information available from ecological studies of NTDs and other parasites of humans and livestock, a direct assessment of the evidence underpinning the hypothesis in figure 1 is challenging. We are nonetheless reminded of the value to be drawn from proxy observations from comparable systems [17]. In terms of parasitology, much of the relevant proxy information has been drawn from wildlife disease ecology, which has tended to pay more attention to the issues of global change than comparable studies on human and domestic animal parasites. In this paper we demonstrate how ecological studies of parasites in wildlife may be used to enhance our understanding of stressors arising from global change, which are likely to be important in the context of parasitic infections (both macro- and microparasites) of humans and domestic animals.

First we consider the influences of some major abiotic and biotic stressors associated with global change and, second, how these stressors might affect parasite life cycles, transmission and ecology. In doing so, we highlight that the abiotic–biotic distinction is blurred, particularly as many stressors also act simultaneously and indirectly on parasites through their hosts. Third, we explore how parasites may respond to the evolutionary pressures of such stressors. Finally, we consider how these complex impacts of global change potentially militate against the sustainable control of parasites affecting animals and humans, and make suggestions for improved understanding and control in an uncertain future world.

## 2. Anthropogenic abiotic and biotic stressors affecting parasite transmission

The majority of modern day ecosystem stressors are driven by industrialization combined with human population growth. These in turn are responsible for increased resource use and generation of waste products, many of which have negative impacts on the environment in complex direct and indirect ways, which may subsequently affect disease risks. For example, the combustion of fossil fuels for energy production and for powering transportation modes significantly contributes to air pollution and carbon dioxide emissions, which promote climate change. Changing land uses, including farming for food, further contribute to climate change and both are considered major drivers of biodiversity loss. Broad-scale biodiversity loss, latitudinal and altitudinal host range expansions and retractions, reduced wildlife population sizes and more limited habitat connectivity are subsequently affecting host interactions and changes in parasite transmission.

### (a) Climate change

The multiple components of climate change, including temperature, precipitation and atmospheric CO_2_, have been extensively studied individually [18–20], but the interactions between these environmental stressors and the consequent effects on parasite transmission are complex. Thus, there is considerable uncertainty about how future climate variation and change will affect disease dynamics [21–23]. Multiple stressors might affect multiple life-history traits, potentially influencing both parasite and host fitness ([24]; see §2). In combination these stressors may counteract each other, such that the overall rate of parasite transmission remains unchanged. Higher temperatures, for example, often increase parasite growth, reproduction and infectivity [25,26], yet can also increase parasite mortality, and as such there is no change in the number of transmitted parasites [27,28]. Likewise, while temperature elevations accelerate the replication of arthropod-borne viruses in their insect or tick vectors, they simultaneously increase vector mortality and decrease biting rate, making the net effect of temperature increase on transmission difficult to predict without detailed knowledge of each component in the system [29]. In other instances, increased temperatures have more pronounced effects on the host, which may exhibit acclimation, adaptation or be forced to shunt resource investment into various life-history components, resulting in thermal preference shifts. Poikilothermic hosts are particularly vulnerable to temperature shifts, but also show remarkable adaptations and such responses by the host can be damaging for the parasite. Some fish, for example, exhibit adaptive behavioural traits to reduce transmission risk, by actively selecting thermal conditions that are detrimental to parasites (behavioural fever; [30]) or selecting flow conditions that minimize fitness costs of infection and potentially reduce transmission [31,32].

Disentangling anthropogenic environmental change from that of natural variation is problematic, particularly for indirect effects and naturally rare events such as extreme weather conditions or disease outbreaks [33,34]. The relationship between environment and transmission is also complex. Different environmental parameters may have additive, multiplicative or antagonistic and nonlinear effects on transmission, which themselves may be intercorrelated or vary at different spatial or temporal scales, with such effects difficult to measure [35,36]. Such relationships may be a consequence of transmission mode. For example, flooding events can be a key driver of some water-borne disease epidemics [37], while drought conditions cause hosts to aggregate at sites where water is available, amplifying transmission and triggering outbreaks of vector-borne diseases such as African horse sickness and Rift Valley fever [38,39]. Other environment–transmission relationships are likely to be a result of a host–parasite range shift due to climate warming. This can change the distribution of vector-borne diseases, including malaria [40] and Rift Valley fever [41]. However, climate change is not spatially homogeneous and could render previously suitable areas unsuitable for transmission and vice versa [42]. The effect of range shift can be yet more complex if the degree or rate of change differs between the host and parasite, causing host–parasite interactions to decouple across some or all regions [43]. For example, tick-borne encephalitis virus (TBEV) transmission is sustained only when temperatures result in synchronous feeding of larvae and nymphs [44]. Projected temperature rises might desynchronize feeding and shrink the area within which TBEV persists [45]. Even the immediate effects of change in temperature and rainfall on parasites are therefore complex and strongly modified by host factors.

### (b) Pollution

Pollutants can cause sublethal physiological stress to hosts and hence reduce their capacity to withstand parasite invasion and/or proliferation, potentially increasing infection levels indirectly (e.g. [46]). However, pollutants also impact parasites themselves, and in aquatic ecosystems, both the infective stages of parasites and their intermediate hosts can be highly sensitive to their effects [47]. Heavy metals can inhibit the release of trematode cercariae from molluscan hosts, as well as impair their swimming behaviour and longevity [48–50]. Pharmaceutical pollutants are widespread stressors likely to affect host susceptibility to disease. The scale of this threat is increasingly apparent in aquaculture: in Chilean salmon farms alone, hundreds of tonnes of antibiotics are used annually [51]. Eutrophication—another important stressor of aquatic ecosystems, arising from excessive nutrient input—is associated with elevated intermediate host densities, parasite fecundity and increased prevalence of certain pathogen infections [52]. However, as yet, there is no overall consensus on its consequences for general patterns of infection [53,54].

Other forms of pollution are less well studied with regard to disease transmission. While it is known that light pollution can impact the structure and function of ecosystems via cascading effects [55], and that natural light cycles govern both relevant parasite life-history traits (e.g. egg hatching; [56]) and intermediate host behaviours (e.g. zooplankton diel migration; [57]), studies on the effects of light pollution on human parasite transmission remain limited [58]. Although the introduction of electricity to socio-economically developing communities has overall human health benefits, night lighting inevitably attracts certain insect vectors and increases human night-time activity. Thus vulnerability to being bitten by a vector is increased, which is implicated in higher incidences of leishmaniasis and malaria in some regions [58]. In other insect-vectored diseases, artificial lighting may have a less overt effect on transmission dynamics: triatome bugs, the vectors of Chagas' disease, typically avoid well-lit areas and artificial lighting may be driving Chagas transmission towards a sylvatic cycle [58]. Noise pollution, a known stress-induced modulator of the immune response [59] that can significantly affect behaviour and predator–prey interactions [60], has not yet been considered in terms of infectious diseases, even though it could have a major influence on farmed animals. The gaps in knowledge concerning the impacts of all types of pollution on parasite transmission are considerable, and without this information it is challenging to assess its importance across host–parasite systems.

### (c) Habitat loss and fragmentation

Habitat alteration due to climate change or anthropogenic activity poses a major threat to ecosystems, often leading to substantial loss of biodiversity, ecosystem functioning and services, and reduced resilience to external stressors [61–65]. This in turn may alter host–parasite interactions, by either increasing [66–69] or decreasing [70–72] infection levels, depending on nuances of host and parasite life history (see §3). The effects of habitat change can even have contrasting effects on closely related parasite species infecting the same host; for example, sunbirds in disturbed habitats exhibited increased prevalence of *Plasmodium lucens* but decreased prevalence of *P. megaglobularis* [71]. Habitat loss and fragmentation also increase the frequency of ‘edges’—transition zones between habitats [73,74]—which are typically exposed to more extreme climatic conditions than interior sites [74]. Habitat edges often promote increased species diversity (i.e. the richness and/or relative abundances of species [75]), resulting in altered levels of interspecific competition and parasitism [76–79]. How the differential effects of edge versus interior sites impacts parasitism varies between host–parasite systems; infections may significantly increase [77], decrease [78] or be unaffected [80]. Although re-establishing connectivity may facilitate initial disease spread [81], in the long term, a larger host gene pool is likely to decrease vulnerability to disease [82], while also increasing overall biodiversity.

### (d) Host density and farming intensification

Over the past 50 years, there have been unprecedented changes in farming practices and associated land use [83]. Although natural and managed forestry currently occupies about 30% of total land area, the impact of deforestation and land use intensification, especially on soil degradation, is significant. Growth in crop production and livestock has been driven by the demand for higher yields. Livestock production is the largest user of agricultural land, accounting for more than 30% of the earth's ice-free terrestrial area [83,84], but aquaculture is the fastest growing food sector [85]. Modern and large-scale farming practices typically rely on concentrating and containing inbred hosts, which can increase host exposure to and facilitate parasite transmission [86,87]. High host density is particularly important for tick-borne pathogens [88], as these vectors are relatively immobile and host–parasite contact frequencies tend to be driven by changes in host abundance and/or behaviour. Chronic stress induced by high stocking densities in aquaculture can have important implications for fish immuno-competence [89], but relationships with infection levels are variable. While high host densities can promote greater parasite population densities, the number of conspecific parasites per host may be reduced [90]. This ‘dilution effect’ (see §2f) is illustrated by a reduction in directly transmitted sea lice at the high host densities in salmonid cage aquaculture [91]. Positive effects of high host density on transmission can be attenuated by mixing susceptible and resistant hosts in rotational grazing systems [92], showing the importance of multiple hosts in modifying infection pressure. However, in aquaculture, where hundreds of thousands of hosts are contained together, this is not yet possible [93], partly because of the need to track farmed fish in the event of an accidental release, and also because of concerns about disease transmission between farmed and wild stocks (and vice versa).

### (e) Urbanization

While density-dependent transmission of human parasites may be expected to increase with high population densities and ownership of companion animals, decreased human–wildlife contact and better sanitation in cities of developed countries generally point to lower levels of disease transmission among such populations (e.g. [94]), although there are exceptions. Dengue, for example, is more prevalent in urban areas due to the provision of suitable human-created microhabitats for the *Aedes* mosquito [95]. Urban environments with high human densities are potentially more vulnerable to water-borne or faeco-orally transmitted parasites if investment in sanitation infrastructure is neglected or disrupted due to socio-economic unrest. Poverty is an important related factor; a study of the contiguous cities of Laredo (USA) and Nuevo Laredo (Mexico) on the USA–Mexican border found that dengue transmission was strongly affected by income, and hence access to technologies such as air conditioning [96]. In developing countries, human–wildlife conflicts can be a major issue. Most emerging and re-emerging human infectious diseases (EIDs) are zoonotic, typically with origins in mammalian wildlife [97,98] or interactions between wildlife and domestic animals [99,100]. This might increase further as habitat loss forces the co-occurrence of wildlife and humans, although this could be offset by the greater effects of biodiversity loss (see below).

A major factor underpinning urbanization is demographic change. By 2050, it is estimated that almost half the world's population will live in the tropics, of which approximately 66% are likely to be living in urban contexts [101,102]. Millions of individuals are also expected to migrate during their lifetimes due to factors associated with the urban–rural cycle, extreme weather events, economic necessity, water and food security, and conflict [103]. Increased patchiness of wealth associated with urbanization, combined with disrupted social structures has already changed the entire landscape of NTDs. These diseases are no longer exclusively prevalent in less developed countries; instead they infiltrate impoverished areas of all countries, including those in the G20, giving rise to the global pattern of ‘Blue Marble Health’ [104].

Associated with urbanization is increased road building. Approximately 60% more roads are projected by 2050 compared with 2010, mostly in developing countries [105], potentially making road building one of the most significant drivers of future environmental change [106]. Road building has already increased the risk of some diseases associated with human development (e.g. agricultural intensification), with an increase in the number of human hantavirus cases reported following completion of a highway through the Brazilian Amazon [107]. Such large-scale road building will almost certainly further facilitate bushmeat hunting in the most biodiverse regions of the planet [108] and change the scale at which people are able to move wild animals out of newly exploited areas and into commodity chains, thereby increasing public health zoonosis risks.

Overall, pathogens likely to thrive as a result of urbanization tend to be either those for which transmission is strongly density-dependent, or those with vectors or reservoirs that are themselves well adapted to urban environments. The net effect on parasite burdens will be highly case-specific and difficult to predict, especially where urbanization is rapid and strong interactions with rural populations persist [109].

### (f) Biodiversity loss

Current extinction rates are estimated to be 100–1000 times greater than background levels [110], with biodiversity loss being one of the hallmarks of the Anthropocene [111]. Loss of host diversity can reduce disease risk directly or indirectly through the associated loss of parasite diversity [112]. For example, reduced risk of African sleeping sickness in humans [113] has been related to the loss of wildlife host biodiversity (reviewed in [114]). Wildlife biodiversity is often correlated with human infectious disease risks. Examples include correlations between mammalian biodiversity and global biogeographic patterns of human infectious diseases [115], elevated likelihood of observing emerging infectious diseases [97] and increases in human pathogen richness and prevalence for some diseases [116]. However, in these cases it can be difficult to separate cause from correlation as areas with high levels of biodiversity are also characterized by other, unrelated, risk factors for disease transmission such as climate and poverty. Nevertheless, the fact that most human infectious diseases have origins in animals, mostly wildlife, supports suggestions that these correlations are mechanistically reasonable and that one large-scale consequence of biodiversity loss could be an overall reduction of disease transmission.

Wildlife biodiversity loss can, however, also increase disease risk. In some ecosystems the number of transmission-competent hosts is ‘diluted’ by abundant non-competent hosts, so the chance of a vector feeding on a suitable host, or of a motile infective parasite successfully contacting a transmission-competent host, may be reduced. When members of a host community are lost due to habitat loss, for example, the risk of disease to a focal host (e.g. humans) could rise. This appears to be the case for Lyme disease in North America [117–119] and there is support for generality across multiple systems [120]. In addition, generalist host species that cope more effectively with human pressure may exhibit greater reservoir competence, or the capacity to transmit pathogens, such that biodiversity loss could select for species that contribute to higher levels of parasitism [121]. Nevertheless, many studies continue to demonstrate that the dilution effect is likely to be of limited generality [122–125], and the net contributory effect of biodiversity (and its loss) to disease risks requires the balance of costs and benefits to be more thoroughly and objectively addressed [126,127]. The notion that wildlife biodiversity can provide an important service in regulating the risk of infectious disease is attractive and has received widespread exposure, although because the interactions that result in transmission events can be complex, the evidence for widespread effects continues to be mixed. In many cases, community composition including relative abundance, rather than biodiversity loss, is a greater predictor of disease risk dilution [122–125].

Biodiversity loss, and its implications for disease risks, may also be experienced at the individual host scale, with subsequent impacts on micro- and macroparasitic infection and transmission. All multicelled organisms are colonized internally and externally by communities of bacteria, eukaryotes, archaea and viruses [128]. These microbiota play a critical role in host health, particularly the gut microbiota and its involvement in immune system development and function [129,130]. In vectors such as *Anopheles* mosquitoes and triatome bugs, an enriched midgut microbiota stimulates upregulation of immune genes that inhibit microparasite development; however, reduced microbiota diversity arising from direct antibiotic treatment or by ingestion of antibiotics circulating in a blood meal is associated with increased microparasite infection of the insect host [131–133]. Moreover, microbiota depletion increases survival and fecundity of the vector itself, potentially exacerbating microparasite transmission [133].

The effects of anthropogenic stressors and within-host biodiversity loss on enteric helminths are highly species-dependent. Certain antibiotics remove *Syphacia* pinworms and other gut helminths in laboratory mice as a direct effect on the parasites themselves or through altering microbial composition, yet other antibiotics have the opposite effect on *Aspiculuris* pinworms, with treated hosts harbouring nearly twice as many worms as controls (reviewed in [134]). Similarly, there are direct links between the loss of bacterial diversity and truncation of helminth life cycles. Eggs of the hookworm *Trichuris muris* require a structural component of Gram-negative bacteria from the host's gut to trigger a signal transduction cascade to stimulate hatching [135]. The nematode *Heligmosomoides polygyrus bakeri* exhibits bacterial dependence for larval development; reared in axenic conditions, the nematodes do not survive beyond second-stage larvae [136]. This suggests that transmission of both *T. muris* and *H. polygyrus* is unlikely to be successful if gut microbiota diversity is inadequate, though confirmation is required from *in vivo* studies. These examples illustrate the potential importance of internal and external biodiversity to parasite transmission and maintenance, and support the notion of biodiversity loss being more far-reaching than is currently recognized.

### (g) Altered interspecific interactions

Changes in host interactions, often linked to the stressors listed above, can drive disease emergence in new hosts. We have already highlighted this problem in association with increased human–wildlife contact, but this in turn might be altered by a range of non-human interactions. Parasites have a fundamental role in food webs [137,138]; thus, anthropogenic changes that reduce the density of higher trophic-level species that feed on larval parasite stages [139] could directly increase disease transmission to competent hosts. Parasites may also indirectly disrupt predator–prey interactions [140] and abiotic factors may affect trophic transmission by altering host foraging activity [26,141]. In addition to these altered parasite–predator–prey interactions, parasites can affect native–invasive host interactions (e.g. [142,143]); newly invading hosts either bring with them novel pathogens to which native hosts do not have resistance, or—having escaped their own native parasites—they can dilute the pool of susceptible native hosts [144].

Finally, parasite–parasite interactions affect transmission, with many studies highlighting the complex interactions of co-infecting parasites in wildlife (e.g. [145]) and livestock (e.g. [146]). As well as parasites having their own microbiota, they can serve as hosts for hyperparasites, the occurrence and life history of which are likely to be influenced by environmental changes [141,147]. Abiotic or biotic stressors may even drive symbionts to adopt parasitism, for example, where there is high competition on the host (e.g. [148]). Artificial manipulation of species interactions can be used in biocontrol, as in the case of *Wolbachia* infection of mosquitoes, which reduces their vectoring capacity [149].

### (h) Interacting abiotic and biotic factors

The above list is not comprehensive, but rather highlights some of the key abiotic and biotic factors that may act together as ‘cocktails’ of stress, with implications for increasing or decreasing disease risks. Identifying the direct and/or indirect factors responsible for changes in disease risk is challenging because multiple stressors act simultaneously on both parasites and their hosts. Depending on habitat and season, the peak impact of different abiotic stressors can occur in or out of phase with one another; thus, while some organisms may be exposed to multiple stressors simultaneously, others will experience them sequentially. Yet, the consequences of multiple, interacting environmental threats for parasite transmission remain unclear: when they co-occur temporally and spatially, their combined effects may be additive, antagonistic or synergistic [150,151]. For example, while elevated seawater temperatures increase mortality rates of oyster larvae, this can be offset by simultaneous water acidification, which reduces the growth of pathogenic bacterial infections [152]. On coral reefs, the interaction between ocean acidification and warming contributes to coral bleaching and reduced disease resistance, leading to increased pathogenicity of existing pathogens and the emergence of new diseases [153]. These two examples are rare, because compared with terrestrial and freshwater systems, marine systems are often neglected with regard to assessing the impact of environmental stressors [154].

## 3. How might parasite life-history traits modulate responses to abiotic and biotic stressors?

Given the complexity of the possible effects of global change on parasite transmission, understanding the factors that drive responses across parasite taxa is essential for more general predictive ability. Here we consider the variety and complexity of parasite life cycles, as the number and diversity of hosts underpin not only how parasites might respond to environmental change, but also their relative fitness and resilience to environmental change at different life stages [155].

Parasite life cycles exhibit remarkable diversity in form and complexity. Whereas some parasites can complete their life cycle infecting a single host organism, others must negotiate their way through several host species in a particular sequence in order to achieve reproductive success. Life cycles with greater complexity rely on biodiverse and integrated communities, and as such may be highly sensitive to the loss of individual components, in the form of host, vector or species interactions required for transmission [112,156]. The level of life cycle flexibility and host specificity is also likely to influence the sensitivity of parasites to changing environments, and their ability to prosper in perturbed ecosystems.

### (a) Life cycle flexibility

The use of paratenic hosts, which are not necessary for parasite development but can sustain parasites and make them available to subsequent obligate hosts, may positively influence transmission if environments become unsuitable or if non-native species outcompete and drive native obligate intermediate hosts locally extinct [157]. An example is provided by two sister species of *Bothriocephalus* cestode, of which only one (*B. gregarius*) uses a facultative paratenic host. Whereas paratenic hosts enhance the probability of *B. gregarius* successfully infecting definitive host fish, resource competition within paratenic hosts lowers infection intensities, and smaller progeny are produced relative to *B. barbatus* [157]*.* Consequently, reduced energy expenditure on growth enables *B. gregarius* to invest more in reproduction and dispersal, increasing the likelihood of re-establishment in a new population of intermediate hosts [157–159]. Alternatively, if populations of the definitive host of *B. gregarius* were to rapidly decline, the paratenic host might potentially replace this host [157].

Parasites that have the capacity to truncate their life cycle may be advantaged under fluctuating environments [160]. For instance, if an obligate host is temporarily unavailable due to seasonally induced migration or anthropogenic activity, developmental requirements for the absent host would be disadvantageous [161,162]. Flexibility in host use may, therefore, allow parasites to cope with seasonal variation in host availability; for example, *Gymnophallus choledochus* normally employs a three-host cycle in summer, but switches to a two-host cycle during winter [163]. In other species, a host may be lost permanently due to strong selection pressures, such as the lack of predators facilitating onward trophic transmission; this ‘missing host hypothesis’ could explain the two-host life cycle of schistosomes [161]. In a more extreme example, *Mesostephanus haliasturis* can forgo sexual reproduction by completing development, via asexual reproduction, in its snail host [164].

Other parasites employ progenesis (precocious sexual maturation) to shorten their life cycle. For example, host diet and increased temperature can induce progenesis in *Stegodexamine anguillae* metacercariae via secreted host-stress signals [160–162]. Thus progenesis may benefit immature trematodes when transmission to definitive hosts is compromised by the health of an intermediate host [162]. For other parasites, such as the hyperviviparous gyrodactylids, progenesis is the norm, and the first-born offspring always develops asexually from the parental worm while it is still an embryo [165]. This adaptation, together with a direct life cycle, facilitates invasion of new habitats: a host only needs to be infected with a single *Gyrodactylus* worm to initiate an epidemic [166].

Life cycle plasticity is particularly advantageous when facing increasing environmental and host uncertainty. Monogenean parasites of the genus *Polystoma*, which typically infect the urinary bladder of frogs, exhibit life cycle dimorphism, with parasites maturing in either three weeks or three years [167,168]; precocious maturation (neoteny) on the tadpole gills occurs when environmental conditions are unsuitable for normal development [169]. Although release of eggs from the slower-growing bladder morphs is induced by the mature host's gonadotropin secretions during the breeding season, both the timing of parasite egg hatching and tadpole development are sensitive to ambient temperatures and chemical environments [170]. Disrupted host chemical balance and light intensity, for instance caused by pollution, may shift the equilibrium between parasite morphs to favour either the neotenic or the slow-growing phenotype [169,170]. Similarly, phenotypic plasticity in the life cycle of the common dog parasite, *Toxocara canis* is dependent upon the physiological status of the host: patent infections develop only in young dogs, while larvae arrest in older hosts and are only reactivated in bitches [171], though host drug treatment might have a hidden influence.

### (b) Specialist versus generalist life cycles

The evolutionary divergence of parasites has generated varying degrees of specialization in parasite traits within different habitats and hosts, some of which are more likely than others to enhance parasite success in unstable environments [155,172,173]. Although it is logical to predict that generalist parasites are more resilient to global change than specialists [121,174], this is very context-dependent [158] and includes the number of hosts in the life cycle and degree of specificity to each. Furthermore, if global change results in new conditions that are stable, parasites that are locally adapted might develop more specialist tendencies [175].

Zoonotic parasites demonstrate varying degrees of host specificity due to transmission via three, non-mutually exclusive life cycles: sylvatic, domestic and anthroponotic [4,155,176]. Host specialization arises due to parasites' investments towards infectiousness and longevity in particular hosts. For example, the nematodes *Trichinella britovi* and *T. spiralis*, both found throughout Europe, possess sylvatic and domestic (swine) host cycles. However, their epidemiology differs due to their higher adaptability to either swine (*T. spiralis*) or carnivore (*T. britovi*) hosts [4]. Nonetheless, re-establishment of *T. spiralis* in a red fox (*Vulpes vulpes*) population, decades after its elimination from domesticated swine in Northern Ireland, demonstrates how host diversity increases parasite resilience to anthropogenic farming activity; i.e. by providing alternative sylvatic reservoir hosts until preferred domestic hosts become vulnerable to infection [172,177,178].

### (c) Parasite longevity

Parasite lifespan, and the time spent inhabiting different hosts, will influence the susceptibility of parasites to environmental changes, and the type of responses that are most likely to arise. Whereas short-lived parasites with rapid life cycles may be more capable of evolving adaptive response to chronic directional changes in environments, long-lived individuals may be better equipped to withstand acute, transient perturbations. The lifespan of parasitic worms can be hugely variable; among the nematodes it can range from three days in free-living *Rhabdias bufonis* adults to 20 years for *Loa loa* (reviewed by [179]); among cestodes, *Taeniarhynchus saginatus* can live in humans for 35 years [180]; and schistosome lifespans of 20–30 years are documented [181], though the mean longevity in optimal hosts is in the range of five years [182]. Helminth parasites with viviparous reproduction, such as *Gyrodactylus* spp., tend to have the shortest lifespans (few days), with age not only determining reproductive output but also reproductive mode [183]. For all species, the timing of pre-patent and patent periods varies and reproductive output typically declines with parasite age and host status (reviewed by [179]). Aside from the longevity of mature worms, it is essential to consider the persistence and resilience of environmental stages when considering how any particular parasite population will respond to global change.

### (d) Parasite reproductive strategies

Long-lived parasite species tend to be iteroparous (e.g. *L. loa*), while other parasites exhibit semelparity (e.g. the human pinworm *Enterobius vermicularis*). Within a parasite species, timing of reproduction is intricately linked to biological and environmental factors, and for many species transmission is seasonal; in extreme cases this can be incredibly brief. For example, *Polystoma integerrimum* transmission only occurs during the host breeding season [184], and in the related species *Pseudodiplorchis americanus*, transmission can be restricted to just 3 h per year, being entirely dependent on monsoon rains creating suitable habitats [185]. If the rains fail, the adult parasites can reabsorb nutrients from ovoviviparous larvae held *in utero* and transmission is delayed [185], but the long-term implication of this strategy is unknown. Similarly, disrupted weather patterns threaten other seasonally transmitted parasites, such as brood parasitic birds, which risk phenological mismatch with their hosts [186].

Reproductive strategies of endoparasites, in particular, are determined by trade-offs in energy investments against other life-history traits [173]. Schistosomes are the only digeneans whose adult stages are exclusively dioecious and dimorphic [187,188]. Only male *Schistosoma mansoni* retain hermaphroditic traits, implying they are energetically costly, and may have restricted female body-form specialization required for efficient egg dispersal [158,189,190]. Evolution of dioecy in schistosomes via host–parasite coevolution demonstrates resilience to long-term environmental changes; however, slowly evolving adaptations may be disadvantageous in the face of short-term perturbations [187,190]. For both hermaphrodite and dioecious parasites, hybridization provides another tool in the parasite's ability to adapt to changing environments [191].

### (e) Life cycle determinants of global change effects on parasites

Life history theory predicts that while parasites with direct life cycles have fewer energetic restrictions imposed by intermediate hosts and can invest more energy towards growth and reproduction [158,159], their dependence on a single host for reproduction might jeopardize survival. By contrast, indirect life cycles offer increased likelihood of ‘rescue’ for parasites, which may alter host specificity via the addition or exclusion of hosts [157,161,192]. Alternatively, parasites that demonstrate increased specialization of specific developmental stages, such as the dimorphic stages of *Polystoma integerrimum*, can inhabit a wider range of host environments and increase the probability of reproductive success [161,169,170]. Finally, dependence upon specific vectors or intermediate hosts for dispersal and reproduction renders parasites extremely vulnerable to both spatial and temporal climatic changes [2,193,194]. Recent studies suggest that parasite life-history traits may be enhanced by climate shifts and anthropogenic stressors associated with ongoing global change [195], thus providing relatively benign parasites with the potential to become increasingly pathogenic. However, while this is considered a serious threat to wildlife communities already facing mounting population pressures, conclusions are usually derived from assessments of single stressors or single parasite life stages, while the net effects to the parasite and host's whole population are rarely determined [196].

The parameters that characterize the life histories of individual parasite taxa are likely to play a critical role in determining their relative resilience in the face of changing environments. Parasite life cycles range in complexity from direct life cycles with a single host species to those with multiple intermediate and facultative paratenic hosts. The diversity of life cycles and life histories, coupled with variable flexibility and specificity of the parasite, means that there are likely to be winners and losers among parasites in perturbed environments. Whereas increased life cycle complexity might leave indirectly transmitted parasites susceptible to environmental change, if they acutely affect an obligate host population, the existence of multiple intermediate and/or reservoir hosts in a life cycle [162] and facultative paratenic hosts may provide a parasite with greater scope for adaptation [158,197]. Parasite survivorship and fecundity are the two key life history traits that impact parasite fitness, and therefore, transmission. Such traits will be subject to environmental stressors, such as drug exposure, that vary over time [198,199]. In the longer term, where stressors inhibit parasite transmission, they are likely to also impose selection pressure on life-history traits.

## 4. Evolutionary change of parasites

Parasites are perhaps uniquely predisposed to rapid evolution under global change. Not only are effective population sizes large and generation times typically short, but transmission imposes an exceptionally strong filter to exclude maladaptation: infective stages either find a host or die. Genotypes better suited to transmission under particular conditions will presumably be strongly selected for, with unpredictable variation in climate or host availability encouraging genetic diversity and within-genotype flexibility in key life-history traits. The potential for parasites to out-evolve their hosts suggests that increasing, rather than decreasing, parasite risks and burdens will be the norm under global change. However, the complex interactions of current stressors, as discussed thus far, can also act upon parasites at the genetic level, complicating predictions and leading to unexpected future infection patterns. Observations of parasite evolution in response to changing environments in nature are rare, but results from a few example systems are offered here to illustrate the potential diversity of parasite adaptive responses to global change.

### (a) Resilience and plasticity

The complex links between existing environmental variation and disease transmission [200–202] suggest that identifying the impact of anthropogenic activities on the evolutionary responses of parasites over and above natural variation might be challenging. Models predict that increasing seasonal climate variability will drive the evolution of greater resilience of pathogens to environmental fluctuations [35]. This has been demonstrated with more extreme monsoon rainfall patterns linked to a rise in a strain of cholera, which is more resilient to water quality and quantity fluctuations [35]. Similarly, plasticity in parasite traits is likely to evolve in response to increased climatic variability, exemplified by the evolution of a plastic transmission strategy in *Plasmodium relictum* that has seen reproductive rates increase during periods of vector availability, thereby maximizing transmission [203].

Human management of host species and treatment strategies (see §5) are also important drivers of pathogen resilience. For example, selection pressure has resulted in altered strain dominance of the potato cyst nematode *Globodera rostochiensis*. Earlier planting of potatoes to allow growth in months historically too cold for larval invasion is now linked with a faster developing, more fecund strain of the parasite [204]. But by far the most pervasive evolutionary phenomenon due to intervention practices is that of increased drug resistance increasingly seen in parasites of humans [205,206] and livestock [207], including aquaculture species [208,209].

### (b) Infectivity and virulence

Habitat change can strongly influence host–parasite interactions, shifting parasite diversity, abundance and transmission dynamics (discussed in §2). Evolving parasite infectivity and virulence may contribute to factors underlying these observations. Habitat fragmentation leads to smaller, patchier and more isolated populations [3]. In host–parasite interactions, infection and transmission will become more localized under such conditions. Theory and empirical data indicate that this can lead to the evolution of reduced parasite infectivity because of self-shading. This effect arises because, as individual susceptible hosts are rapidly infected locally by virulent parasites, they are surrounded by other infected hosts, which will reduce opportunities for further transmission of horizontally transmitted parasites [210,211] and parasites that use mixed transmission strategies [212]. In contrast with habitat fragmentation, intensification of farming practices is predicted to drive evolution of increased virulence; higher host availability reduces the adaptive cost of increased virulence due to host mortality [93]. Key evidence for this is the recent increase in pathology and mortality due to *Flavobacterium columnare* in densely stocked Finnish freshwater fish farms, linked to the emergence of more virulent, infective strains of the pathogen [213].

### (c) Bet-hedging

Unpredictable conditions, such as the timing of host availability, should favour parasites that can produce offspring that vary in their life-history or transmission strategies [214]. Spreading the risk, or ‘bet-hedging’, allows parasites to increase the chances that at least some of their progeny will survive and infect a competent host. It is reasonable to expect that parasites will increasingly adopt such bet-hedging strategies to ensure survival in rapidly changing environments. The nematode *Nematodirus battus* historically exhibited a single generation per year with overwintered eggs hatching in spring to coincide with arrival of newborn lambs [215,216]. Evolution of multiple generations per year [217] via the production of autumn-hatching eggs that do not require vernalization has mitigated against asynchrony between larval presence and the availability of susceptible hosts in years with early warm springs [218]. However, anthropogenic changes may also hinder the evolution of parasite bet-hedging strategies. Variation in life cycle traits (e.g. rate of development, egg laying and hatching) of the fish louse *Argulus foliaceus* infecting farmed fish is lower than in wild populations, probably as a result of reliable host availability in fish farms compared with natural ecosystems [219].

### (d) Host switching

Global change might constrain host–parasite co-evolution if the benefits of new mutations that enhance fitness (selective sweeps) are not realized in a rapidly changing environment [220]. Alternatively, host switching is a potential parasite adaptation to global change [221,222], should the availability of preferred hosts be decreased via geographical range shifts, phenological asynchrony, human management or control strategies. In some cases, switching to alternative hosts may not be optimal for parasite development, leading to reduced parasite offspring or survivorship and thereby reduced probability of transmission. This may limit how much host switching actually occurs in changing environments. However, Jones *et al*. [223] showed that while costs of prey switching for a parasitoid were severe in the first instance, these costs were ameliorated over successive generations. Furthermore, the force of selection will play a key role in the drive to host switch. In the case of the Guinea worm (*Dracunculus medinensis*), an extremely simple but effective control programme that filtered the copepod vectors from contaminated drinking water effectively blocked transmission [224] and reduced the number of human cases from an estimated 3.5 million cases in 1986 to just 126 in 2014. However, in 2015, 459 infections were recorded for the first time in dogs [13,225], suggesting a potential host-switching event, possibly driven by the effective control measures blocking transmission to humans [225].

The introduction of invasive host species generates unique opportunities for non-native parasite communities to come into contact with new hosts, and considerable potential for host switching. Classic examples of this include the introduction of squirrel parapoxvirus into UK red squirrels (*Sciurus vulgaris*; [142,226,227]) and crayfish plague (*Aphanomyces astaci*) into European crayfish (*Astacus astacus* [228]). Host switching from introduced to native hosts appears equally common for parasites with direct and indirect life cycles, and worryingly the majority of those reported are more virulent in native hosts than in the co-introduced invasive host [229]; however, considering that we still know very little about parasite speciation, it is difficult to predict future outcomes, and other mechanisms, such as niche specialization [230] and hybridization [231], could also affect both speciation and host range.

### (e) Multiple evolutionary targets for adaptation

The above examples demonstrate how the effects of human activity and climate change are varied and far ranging with respect to parasite evolution. Targets of evolution are already altering the epidemiology of parasites; resilience, strain variation in phenology, bet-hedging in key life-history traits and host switching all demonstrate that through past unpredictability in transmission, parasites are well adapted to future changes in climate and host availability. As the evidence for the anthropogenic effects on parasite adaptive responses builds, we must now consider the evolutionary capabilities of pathogens as an integral component to predicting the future landscape of host–parasite interactions under pressures of global change. This will be particularly important when considering the consequences of parasite control programmes, arguably the greatest selective pressure faced by parasites in their evolutionary history.

## 5. Control programmes and predictive epidemiology in a changing world

Taken together, the observations and projections described above give a strong signal to all epidemiologists: the future is both uncertain and rapidly changing, representing a new era of health challenges in the twenty-first century that is unprecedented in human history. Multiple laboratory and field observations, modelling exercises and meta-analyses have identified key abiotic and biotic factors that govern the free-living and vector-borne stages of parasites (see §2). Seasonally variable environments are also important in determining the aggregation of animal parasites (e.g. [232]) including malaria and hookworms in some regions [233,234]. What remains unknown is how patterns of global change across decadal scales have influenced parasite transmission. While the substantial post-1997 downturn in malarial infections has undoubtedly been accelerated by large-scale control interventions, environmental changes that have reduced the vector population or climate-sensitive parasite life stages over extended periods may have also contributed. Droughts in Africa are increasingly common [235], and we cannot exclude the possibility that prolonged periods of low rainfall have contributed to the downturn in transmission of malaria and other parasitic infections, given the reliance of vectors and parasite transmission stages on water availability. This last point illustrates how cryptic factors continue to be influential. Most campaigns do not routinely collect individual patient data once the delivery programme is established, and without this it is not easily possible to differentiate the effects of MDA from those of environmental change.

### (a) Evolutionary implications of control programmes

MDA programmes themselves are stressors on host–parasite systems as a result of imposing selection pressures, altering host microbiota and disrupting life cycles of vectors and intermediate hosts that have coevolved with parasites. Thus the success of these programmes should also be considered in the light of changing abiotic and biotic factors. The impetus for MDA campaigns stems from mathematical models of parasite life cycles first developed in the second half of the twentieth century (e.g. [236]), which have an underlying assumption that neither parasites nor vectors undergo significant evolution that would reduce the impact of intervention. More recent attempts that consider either diminished drug efficacy and/or greater transmission rate lead to the common output that MDA programmes do not eliminate parasite populations even after decades of intervention. Evolution of the parasite and/or vector populations along a specific life-history trajectory has not been a common feature of these models.

Long-term control programmes may do more harm than good, as any treatment imposes a selection pressure for resistance on the parasite population. Some treatments may promote the evolution of more virulent pathogen strains [237] as illustrated by evolving rodent malaria parasites in mice immunized with a candidate human malaria vaccine [238]. This in turn might lead to enhanced transmission as shown in chickens vaccinated against Marek's disease virus [239]. Poulin [198] suggests that shortening the duration of parasite infection by drug treatment negates any advantage to a parasite being long-lived, and survivorship of a parasite may therefore trade-off against other traits that affect infectivity, such as age to maturity and fecundity (see §2), resulting in enhanced transmission. Virulence–longevity trade-offs might explain increased horizontal transmission of some diseases on hospital wards [240], while nematodes of horses appear to respond to drug treatment by shortening their development period in the host [241]. According to Day & Read [242], the optimal approach for combating the evolution of drug resistance is to use the highest safe dose or the lowest, effective dose. High-dose medications are effective only if all pathogens can be killed (as in the case of HIV). If a small number of microbes are likely to evade treatment (already resistant to treatment, or if drug resistance arises *de novo*), then high doses of medication may allow resistant microbes to survive and spread by the very act of killing off drug-sensitive microbes [242]. On the other hand, low drug doses are more likely to enable new mutations conferring drug resistance to spread and fix in the parasite population. Whether high or low doses are optimal for combating drug resistance will depend on system-specific factors, but this study highlights that parasite evolution is rarely considered proactively in setting treatment goals and decisions.

One consequence of the ‘perfect storm’ of stressors may be the generation of so-called ‘hotspots’ of transmission [243]. This hypothetical effect does not preclude a reduction in transmission intensity, but does imply that individuals who are normally resident in areas of intervention are persistently exposed and harbouring infection. Other interpretations of the hotspot observations are possible—including the lack of engagement or disenfranchisement with control programmes [244].

### (b) Adaptive management and predictive epidemiology

Changes in parasite ecology, epidemiology (§§2 and 3) and evolution (§4) have profound implications for the monitoring and control of health in managed systems, which must themselves adapt if altered challenges are to be attenuated. To keep pace, we need to develop a predictive understanding of how patterns of parasite transmission among animals and humans could change in response to the multiple, interacting stressors being placed on the global ecosystem [245]. From this understanding we need to create improved decision support systems that allow for sustainable control and management of hosts, vectors and parasites. However, the wide range of relevant anthropogenic stressors, the enormous diversity of parasite taxa, life-history traits and infection strategies, and the range of possible functional responses and interactions between them—coupled with simultaneous responses among host populations—make this a hugely challenging task. Here, we offer one approach based on co-production, knowledge transfer and wider participation.

The subtle, ‘covert’ ways in which multiple drivers of global change can affect parasite transmission are complex when considering individual stressors, while the impact of interacting stressors on future disease risk remains largely unknown. The current practice of making iterative changes in management strategy, based on accumulated evidence of infection patterns, is too static to keep up with the increasing uncertainty around transmission patterns. At the same time, advances in information and communication technology open up new data collection modalities. Organizing and applying such data streams could provide novel and powerful ways of gathering real-time understanding of changing transmission, and adapting control practices accordingly.

The zenith of adaptive management would track and react to not only parasite transmission but also evolutionary processes, including those of host populations, such that transmission functions are re-evaluated as life-history parameters change (see §§ 3 and 4 above). This would require repeated confrontation of alternative transmission models with available data, and inferring shifts in key parameters from model fits. In principle, this is already possible, but automation of the process and the availability of sufficient, robust and timely data present challenges to implementation. Citizen Science has been leveraged to gather real-time information on the distribution of invasive plant diseases [246], while mobile phone networks have been used effectively to gather data on the changing epidemiology of diseases in livestock and humans [106,247,248]. Involved professionals such as farmers and veterinary laboratories are also a source of specific surveillance data [249], which could be collated more quickly to track epidemiological patterns, and update models accordingly. For example, confirmed diagnoses of infection with the helminth *N. battus* in lambs are currently used to populate web-accessible maps that are updated daily during high-risk periods (www.scops.org.uk). Linking these data with models of parasite transmission dynamics and life-history parameters modified according to observations could make such models robust to parasite evolution (e.g. bet-hedging; see §4c), even pending more complete knowledge of how evolution alters epidemiology.

A major challenge in dynamic data-driven model fitting is the reliability of data collected from disparate sources, and not always by professionals using verified methods. However, the decline of expensive, centrally funded monitoring stations and systems, both for meteorological and disease data, limits the alternatives. Networks of privately collected meteorological data (e.g. forecast.io) are increasingly available and may usefully compensate for the loss of, and sometimes exceed the capability of, official sources. Similar networks for data on phenology or even parasitic infections could be envisaged. Separate observational or experimental data will always be needed and can constrain fitted parameters within plausible ranges, or select parameters most likely to be open to parasite evolution. Models of parasite transmission dynamics that are validated, updated with shifts in epidemiology and evolution, and whose outputs are accessible to end users, could form the backbone of a new wave of decision support systems that maximize the opportunities afforded by advances in modelling and new sources of data. In addition, by involving the public in disease monitoring, we can promote disease awareness, improving socio-ecological resilience [250].

We suggest that a truly predictive understanding of the effects of global change on parasite transmission will, therefore, need to incorporate the evolutionary consequences of changes imposed by combinations of abiotic and biotic stressors acting at various locations under conditions of migration, habitat loss and fragmentation. These are themselves difficult to predict, especially as the experiments required to fuel such predictions would tend to remove ‘extraneous’ variation that could actually be core to complex evolutionary trajectories under global change. Reverse engineering of models to estimate parameter alterations, which are needed to maintain parasite fitness under changing scenarios, and subsequently assessing these predictions for biological plausibility, could provide more adaptive predictions. In any case, models of parasite population dynamics that neglect the possibility of evolution of transmission strategies will have a short shelf-life under global change, and greater attention ought to be paid to this challenging area.

## 6. Conclusion

Differences in transmission ecology, parasite life history and the ecology of intermediate hosts and vectors will clearly play a key role in determining the sensitivity of infections to abiotic and biotic stressors [202]. Monitoring these stressors at high spatial and temporal resolution, perhaps using remotely sensed products (e.g. [251]), is likely to be of considerable help in improving our understanding of how diseases might spread in the future. However, while there is a move away from using keystone species in the general ecological field as early warning indicators of vulnerable ecosystems in favour of monitoring the balance between diversity, functional groups and connectivity, it would be naive to take this approach for infectious diseases. The impact of infectious diseases, particularly EIDs, is context-dependent (the devil is in the detail) and the importance of particular parasite species and strains will change over space and time, and so at least for the moment, targeted disease monitoring and surveillance at appropriate spatio-temporal resolution are still necessary.

## Glossary

term: definition
parasitological terms
definitive host: Host in or on which a parasite reaches sexual maturity.
ectoparasite: Parasite that lives on the exterior of a host.
endoparasite: Parasite that lives inside the body of a host.
helminth: Parasitic worm from either the Phylum Plathyhelminthes or Nematoda.
intermediate host: Host required for larval development or growth of a parasite before it is infective to another intermediate host or the definitive host.
macroparasite: Parasite that produces infective stages released away from the host; typically of a larger body size than a microparasite.
microparasite: Parasite that reproduces *in situ* on the host; typically microbes (viruses, bacteria, protozoa) but exceptions include viviparous helminths, such as *Gyrodactylus* spp.
parasite: Organism that lives at the expense of its host and causes harm; includes both micro- and macroparasites.
parasite intensity: Number of individual parasites of a particular species infecting a host.
prevalence: Number of individuals of a host species infected with a species of parasite. Expressed as a percentage.
systems biology terms
global change: Any climatic or anthropogenic change that has an impact directly or indirectly on an ecosystem; includes all stressors associated with urbanization and human population growth (growth of urban areas, depletion of rural and natural resources, etc.).
industrialization: Social and economic change that has transformed human societies from agrarian to industrial, resulting in urbanization, exploitation of natural resources and environmental pollution.
planetary boundaries: Framework for identifying a ‘safe operating space for humanity’ covering nine earth system processes. Each process has a tipping point demarcating safe zones [6,7].
social–ecological resilience: Capacity of an integrated ecosystem that includes humans to withstand perturbations and stressors.
systems change: Biological, social and political drivers, affecting interactions, functioning and dynamic processes within ecosystems.
urbanization: Increased numbers of people in towns and cities resulting in growth of urban areas.
